# Reliability, validity and normal ranges of the Zur Balance Scale for detecting mild postural control differences: introducing the modified, short version mZBS

**DOI:** 10.3389/fnhum.2023.1131478

**Published:** 2023-05-26

**Authors:** Oz Zur, Hadas Ben-Rubi Shimron, Lisa Deutsch, Eli Carmeli

**Affiliations:** ^1^The Israeli Center for Dizziness and Balance Disorders, Ra’anana, Israel; ^2^Department of Physical Therapy, Ben Gurion University of the Negev, Be’er Sheva, Israel; ^3^BioStats Statistical Consulting, Ltd., Modi’in-Maccabim-Re’ut, Israel; ^4^Department of Physical Therapy, University of Haifa, Haifa, Israel

**Keywords:** balance, postural control, vestibular, sensory integration, kinetics

## Abstract

**Introduction:**

Balance is achieved through interactions between the vestibular, somatosensory, and visual systems. There are several clinical tests to measure postural stability. However, most of them do not assess postural stability with head movements, which is the main function of the vestibular system, and those that do, require the use of sizeable, expensive equipment. Therefore, an applicable, easy-to-perform test that challenges the function of the visual, somatosensory and vestibular systems, using head movements, is needed. The Zur Balance Scale (ZBS) contains ten conditions, which are a combination of surfaces (floor or Styrofoam with subject standing on its width in Romberg position or its length in tandem position), stances (Romberg or tandem), tasks (no head movement with eyes open or closed and horizontal or vertical head movements with eyes open). The purpose of this study was to determine the validity, inter- and intra-examiner reliability, and normal performance values of the ZBS among individuals 29–70-years of age and to introduce the modified version: the mZBS, using kinetic measurements.

**Methods:**

Healthy participants ages 29–70 years were evaluated for inter- and intra-tester reliability (*n* = 65), kinetic measurements on a force plate, and validity compared to the modified clinical test of sensory interaction and balance (mCTSIB) (*n* = 44) and characterization of normal values (*n* = 251).

**Results:**

Zur Balance Scale head movements, duration of each condition (up to 10 s) and the total ZBS score agreed across examiners (ICC > 0.8). Normal ZBS scores were negatively correlated with age (*r* = −0.34; *P* < 0.0001). Older subjects (60–70 years) had a median score of 95.5 compared with younger subjects, where medians ranged from 97.6 to 98.9. Kinetic parameters showed positive correlations between ZBS and the mCTSIB scores, with the highest correlation between the five Romberg tasks (modified ZBS).

**Conclusion:**

Zur Balance Scale is a valid and reliable test. Its advantages include using head movements and the ability to detect minimal differences in postural control, even in healthy populations. Kinetic evaluation of the ZBS enables the use of a modified, shorter version of the ZBS (mZBS).

## 1. Introduction

Balance is achieved through interactions between the vestibular, somatosensory (proprioceptive) and visual systems. Each system contributes to balance. The central nervous system gathers information from the three systems, processes the information and responds accordingly. If one of these three sensory systems is impaired, the remaining systems must compensate for the missing sensory input, to maintain balance. However, none of the sensory systems can completely compensate for dysfunction in another (Simoneau et al., 1995), which makes feedback to the central nervous system less comprehensive, and might impair the precision of balance reactions (Rubenstein, 2006; Sturnieks et al., 2008).

When the vestibular system is not functioning well, one of the most pronounced symptoms is lack of balance. Older adults who present with vestibular deficits are at high risk for falls (Close, 2005; Murray et al., 2005; Zur et al., 2006). The connection between poor balance and risk of falling has been well-established (Zur et al., 2016; Pua et al., 2017; Chen et al., 2021) and proper assessment of balance is an important step in fall prevention and in fall prevention programs (Close, 2005; Ribeiro et al., 2017).

There are many different clinical tests for assessing balance in different age groups. Tests widely used in vestibular clinics include the Berg Balance Scale (Berg, 1989), the timed up and go test (Mathias et al., 1986), the functional reach test, the Mini BESTest, the Fullerton Advanced Balance (Rose et al., 2006), the dynamic gait index (DGI) (Whitney et al., 2004) and the modified clinical test sensory interaction for balance (mCTSIB) (Freeman et al., 2018). These tests identify patients with severe disequilibrium well, but have the disadvantages of a ceiling effect and low specificity for predicting falls (Whitney et al., 2004; Wrisley and Kumar, 2010; Zur et al., 2016). Cohen et al. (2014) reported that adding head movements in vertical and horizontal directions could improve the sensitivity and specificity of the traditional mCTSIB examination performed with the head still. Janc et al. (2021) added head movements to the sensory organization test and had better results with healthy individuals and among people with unilateral vestibular lesions. They reported that adding head movements to the standard static posturography test improved its sensitivity and specificity for vestibular patients. The DGI uses head movements during walking and does not test balance performance in a standing position.

For these reasons, a new balance test that challenges somatosensory systems (vision, somatosensory and vestibular) and uses head movements is needed. The Zur Balance Scale (ZBS) was introduced and tested with a group of participants ages 71–97 years (Zur et al., 2016). It was found reliable compared to the Berg Balance Scale in adults ages 70–95 years. The ZBS evaluates the visual, somatosensory. and vestibular sensory systems that form the basis of postural balance. It focuses on the dynamic function of the vestibular system by using horizontal and vertical head movements during various stances. It requires simple equipment, is quick and easy to administer and analyze.

The main aims of this study were to evaluate the inter-tester and intra-tester reliability of the ZBS and to determine the normative performance scores of the scale for adults 29–70 years of age. The secondary aim was to validate the kinetic data of the ZBS compared to the mCTSIB, using a force plate and to use the precise kinetic measurements to validate a short version of the ZBS (mZBS).

## 2. Materials and methods

This cross-sectional, double-blind study was approved by the Institutional Ethics Committee of Haifa University (approval no. 318, 319/16, date 12/09/2016). All participants signed an informed consent form before participating. Inclusion criteria were ages 29–70 years, healthy, with no known falls or major balance problems in the last 12 months. Exclusion criteria were neurologic, orthopedic or metabolic pathology, or acute vestibulopathy.

### 2.1. Participants

**FIGURE 1:**
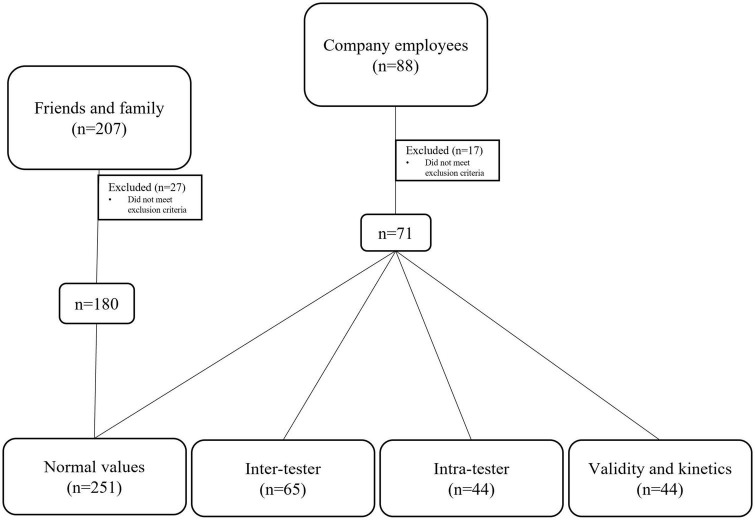
Flow diagram of participant recruitment to the study.

Participants were recruited among employees of a technology company and family and friends. The company employees worked in various departments, including development, administration, laboratories, and accounting. Among the employees, 146 (60%) attended a frontal lecture about balance and the vestibular system. They were told that the general purpose of the study was to measure their balance. Of these, 88 volunteered to participate and signed a consent form. Seventy-one met the inclusion and exclusion criteria and were included in the study to determine inter- and intra-tester reliability, validity, and normative values of the ZBS (Figure 1).

To increase the dataset for determining the normative performance values, an additional 207 family and friends of students studying for a master’s degree in physical therapy were recruited. Among them, 180 met the inclusion and exclusion criteria and were included in the norms analysis only.

Ultimately, 17 participants from the employee group and 27 from the family and friends’ group did not meet the exclusion criteria. Thus, 251 people were included in the study (Figure 1).

### 2.2. Measurement and tools

#### 2.2.1. Zur Balance Scale (ZBS)

Equipment needed to administer the ZBS includes a half-cylinder of Styrofoam (density 30 kg/m^3^, 60 cm long×18 cm wide×9 cm high) covered with a stretchable piece of fabric, and a stopwatch for measuring seconds and milliseconds.

**FIGURE 2:**
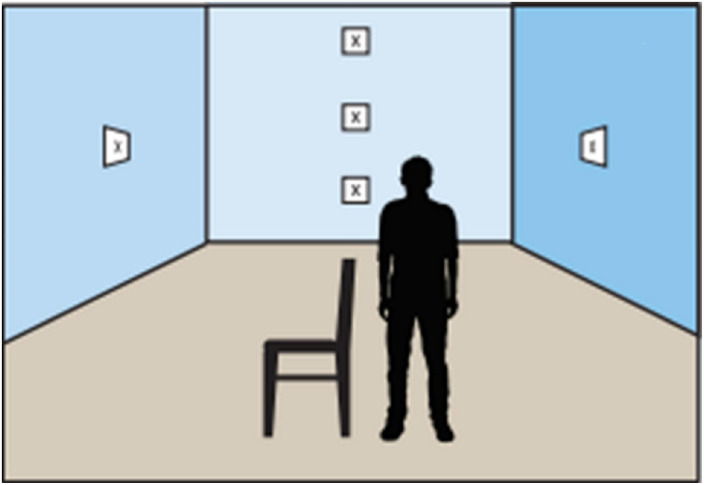
Examination room setting (with permission).

The ZBS is performed in a quiet room. The participant stands two meters from the target–a 5 cm×5 cm X marked at eye level. For safety and confidence, a solid support (like a chair or table) is placed next to the participant (Figure 2), and the examiner stands diagonally in front of the person being tested.

**FIGURE 3:**
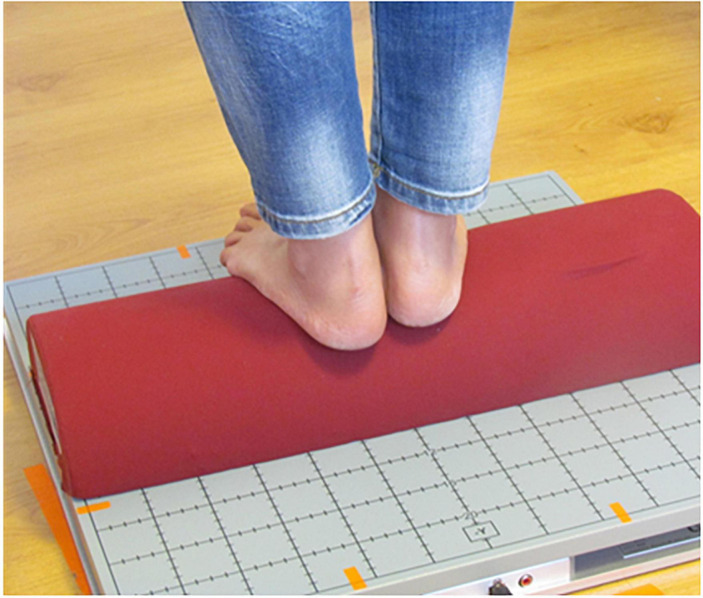
Participant standing on the width of the Styrofoam in Romberg position.

The ZBS contains ten conditions, which are a combination of surfaces [floor, Styrofoam with the subject standing on the width in Romberg position (Figure 3) or its length in tandem position], stances (Romberg or tandem) and tasks (eyes open or closed with no head movement, horizontal or vertical head movements with eyes open).

The ZBS is scored by counting the number of head movements (HM) beginning from one side and time to maintain balance up to 10 s. The time to maintain balance (with and without HM) is measured in seconds, for a maximum of 10 s. The participant may perform each condition twice and the better result is used for analysis. All conditions and the score calculation process are summarized in the ZBS score sheet (Appendix 1).

In 5 of the 10 conditions 2, 3, 6, 8 and 10, the participant is asked to move his/her head left and right, covering an arc of approximately 120° (60° to each side) and a total of 60° up and down (30° up and 30° down) each within 10 s according to a 60 Hz metronome. Zero to 10 HM are performed in each condition, for a maximum of 50. The ZBS score is calculated by summing the total number of HM multiplied by 2 (HM Score), plus the total time (in seconds) for all 10 conditions divided by 2 (total seconds score). The ZBS score is the mean of the head movements score and total seconds scores. Scores can range from 0 to 100 (maximum performance score). For example, 38 head movements multiplied by 2 equals 76 (HM score); total time in this example was 80 s (total seconds score); 76 plus 80 equals 156; 156 divided by 2 equals 78, which is the ZBS score. Using clinical reasoning alone, changes from 5 points and higher in the ZBS total score are meaningful.

#### 2.2.2. Modified clinical test of sensory interaction and balance (mCTSIB)

The clinical test of sensory interaction and balance (CTSIB) was developed to test the influence of visual, vestibular, and somatosensory inputs on balance, using six tasks (Shumway-Cook and Horak, 1986). The modified CTSIB (mCTSIB) was developed to simplify the test and includes only four of the original six tasks (Whitney et al., 2002). The equipment needed to administer this test are Tempur foam and a stopwatch. The test could also be performed on an instrumented force plate to register body sway and kinetic parameters (Whitney et al., 2002; Freeman et al., 2018).

The mCTSIB includes four different conditions where the participant is instructed to maintain static balance for up to 30 s: 1. Stand on a firm surface, eyes open; 2. Stand on a firm surface, eyes closed; 3. Stand on foam surface, eyes open and 4. Stand on foam surface, eyes closed.

The mCTSIB score is calculated by summing the total seconds of all four conditions (max 120 s). For example, condition 1; 30 s, condition 2; 25 s, condition 3; 25 s and condition 4; 20 s. The total score is 30+25+25+20 = 100; which 83% out of 120.

#### 2.2.3. Force plate

A force plate (AMTI, Watertown, MA, USA) is required to evaluate the forces on top of the surface on which the participant stands. Center of pressure data during the ZBS and the mCTSIB were sampled at a frequency of 100 Hz. The following parameters were analyzed using MATLAB software: anterior posterior (A/P) sway, lateral medial (M/L) sway, elliptic sway, and the velocity sway during each of the different conditions or tasks.

### 2.3. Study protocol

To assess inter-tester reliability, 65 participants were evaluated by two physical therapists at different times on the same day. They were asked to perform the ZBS tasks described above, to assess the degree to which different raters agree in their assessments. To determine intra-tester reliability, 44 employees were evaluated twice within 3 days by the same examiner. Validation and kinematic measurements on a force plate are longer protocols; therefore, we tested 44 employees during the 3 days of the study (Figure 1).

Room and daytime conditions were kept identical for each of the three examination days. Examiners were two experienced physical therapists and three students in their final year of study for a bachelor’s degree in physical therapy, who were trained to perform the test. Examiners and participants were blinded to the total score of each examination and the prior tests. The therapists performing the intra-tester measurements could not be blinded to the performance of the participants in the previous test but were blinded to the total ZBS score. All examiners were blinded to the testing order as well. ZBS scores were calculated after data collection, by a separate statistical team.

The 180 family and friends of students were tested in a private clinic under the same conditions. They were tested by nine experienced physical therapists who were students in their last year of a master’s degree program in physical therapy.

### 2.4. Data analysis

Using non-parametric methods, it is impossible to distinguish between two percentiles of a distribution that are P% apart unless at least (100/P)-1 observations are obtained. Thus, to distinguish between the 97.5th percentile and the 95th percentile of the ZBS score and determine reference ranges across age groups (by decade), at least 100/2.5–1 = 39 observations were needed per decade.

Data were analyzed using SAS version 9.4 (SAS Institute, Cary, NC, USA). A *p*-value < 0.05 was considered statistically significant. Categorical data are presented as count and percentage, and continuous data as mean and standard deviation, as well as median and interquartile range (IQR).

Differences between examiners were tested with the Wilcoxon two-sample test. The Wilcoxon sum-rank test was used to compare between sessions with the same examiner. Spearman’s correlation coefficient was used to assess the correlation between age and ZBS scores. ZBS scores were compared between age groups using the Kruskal–Wallis test. Intraclass correlation coefficients (ICC) (as an index of inter-measurement reliability) are presented as measures of within and between tester reliability. ICC coefficients were interpreted in a similar manner to correlation coefficients: we considered an ICC > 0.80 excellent; 0.60 ≤ ICC ≤ 0.80 good; 0.40 ≤ ICC ≤ 0.60 moderate and < 0.40 poor. The minimal detectable change (MDC) (Steffen and Seney, 2008; Hulzinga et al., 2020; Tao et al., 2021; Shao et al., 2022) was calculated using the following formula: MDC = SEM×1:96×$\sqrt{2}$, whereby SEM = SD_*pooled*_×$\sqrt{1-I\,C\,C}$. MDC is defined as the minimal amount of change that is not due to variation in measurement, and can be interpreted clinically as the minimal change that is not due to error.

Zur Balance Scale (ZBS) normal ranges (upper and lower 2.5% percentiles) are presented per age group.

Pearson correlation coefficients are presented to assess the relationship between the ZBS and the mCTSIB with respect to kinematic parameters (A/P, M/L, elliptical sway, and velocity sway), as well as to validate the ZBS test compared with the mCTSIB on a force plate.

We used Cronbach’s alpha to measure internal consistency of the 10 tasks of the ZBS total score and to validate a modified, shorter version of the ZBS comprised of 5 (or fewer) Romberg tasks (1, 2, 3, 7, 8) or 5 Tandem (4, 5, 6, 9, 10) tasks (note that Cronbach’s alpha ≥ 0.70 is considered “acceptable” and ≥ 0.8 is considered “good”).

Zur Balance Scale (ZBS) normal ranges (upper and lower 2.5% percentiles) are presented for the ZBS kinematic parameters (A/P, M/L, elliptical sway, and velocity sway) for all 10 tasks.

## 3. Results

### 3.1. ZBS inter-tester reliability

Sixty-five people participated in the inter-tester reliability portion of the study. Mean age was 47.5 ± 10.3 years. No significant differences were found between the two examiners regarding ZBS head movements, duration of each condition and the total ZBS score. ICC statistics were all > 0.8 and the inter-tester MDC of the total ZBS score was 4.66 points (Table 1). This means that an improvement of 4.66 points in the ZBS score can be detected as not attributed to measurement error.

**TABLE 1:** Difference between two testers in ZBS scores and intertester reliability (*N* = 65).

Task	Mean (SD)	Median (IQR)	*p*-value*	Inter-tester ICC**	MDC^@^
Head movements score, tester 1	96.1 (5.8)	98 (6.0)	0.50	0.81	7.49
Head movements score, tester 2	95.8 (6.7)	100 (6.0)			
Time score (seconds), tester 1	95.9 (4.9)	97.1 (5.1)	0.22	0.84	5.33
Time score (seconds), tester 2	96.4 (4.8)	98 (4.9)			
Total ZBS score, tester 1	96.0 (5.1)	98 (4.9)	0.69	0.90	4.66
Total ZBS score, tester 2	96.1 (5.5)	98.4 (5.3)			

*Wilcoxon-sum-rank test, **intra-class correlation coefficient, ^@^minimal detectable change.

### 3.2. ZBS intra-tester reliability

Forty-four participants from the company group (mean age 47.2 ± 10.4 years) were administered the ZBS twice over 3 days by the same tester, to evaluate the inter-session reliability of the test for stability over time. Both evaluations took place at the same location. The intra-tester reliability of the total ZBS score in terms of the ICC was 0.83 and the intra-tester MDC was 4.92 points. There was no significant difference between the two tests administered by the same tester on different days (Table 2).

**TABLE 2:** Difference between the same tester over time and within tester reliability between scores(*N* = 44).

Tasks scored twice	Mean (SD)	Median (IQR)	*p*-value*	Within tester ICC**	MDC^@^
Head movements first exam	96.4 (5.0)	98 (6.0)	0.80	0.70	7.9
Head movements second exam	96.7 (5.5)	100 (6.0)			
Time first exam, seconds	96.8 (4.1)	98.2 (4.0)	0.26	0.79	5.08
Time second exam, seconds	96.5 (3.8)	97.7 (4.3)			
Total ZBS score first exam	96.6 (4.3)	98.2 (4.9)	0.10	0.83	4.94
Total ZBS score second exam	96.6 (4.4)	98.4 (4.8)			

*Wilcoxon rank-sum test, **intra-class correlation coefficient, ^@^minimal detectable change.

### 3.3. ZBS normal range by age

A total of 251 participants (employees, friends, and family) with no balance problems were included in this evaluation of the ZBS. Mean age was 46 ± 11.5 years and 137 (54.6%) were female. No differences were found between recruited employees and recruited friends and family with respect to age and ZBS score.

**FIGURE 4:**
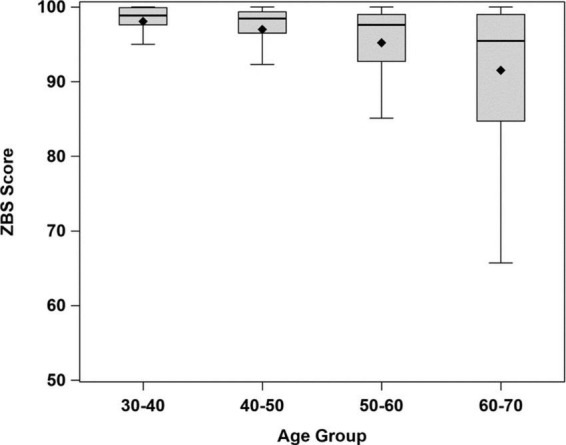
Box plots of distribution of ZBS norm scores by age group (significant difference in ZBS scores between age groups, Kruskal–Wallis test, *p* < 0.0001).

Table 3 demonstrates the normal ZBS scores by age groups. Among all participants, the ZBS scores decreased with increasing age (*r* = −0.34; *P* < 0.001), especially after age 60 (Figure 4).

**TABLE 3:** Zur Balance Scale score distribution in healthy participants and normal ranges of different age groups (*N* = 251).

Age	N	Mean score (SD)	Median (min-max)	Normal range
29–40	98	s98.1 (2.54)	98.9 (88.3–100)	90.0; 100
41–50	68	97.0 (3.8)	98.5 (84.7–100)	85.0; 100
51–60	47	95.2 (6.0)	97.6 (71.0–99.9)	80.0; 100
61–70	38	91.5 (8.8)	95.5 (65.7–99.9)	65.7; 100

### 3.4. Internal reliability and validity of ZBS versus mCTSIB

To determine the correlations between the kinematics (A/P, M/L, elliptical sway and velocity sway) of ZBS and mCTSIB tests, we compared results of the two tests. As a second step, we sought to determine whether a short version of the ZBS could be validated. We compared the kinematics of the mCTSIB with all 10 ZBS tasks, tandem tasks (4, 5, 6, 9, 10) only and all Romberg tasks (1, 2, 3, 7, 8) (Appendix 1).

**FIGURE 5:**
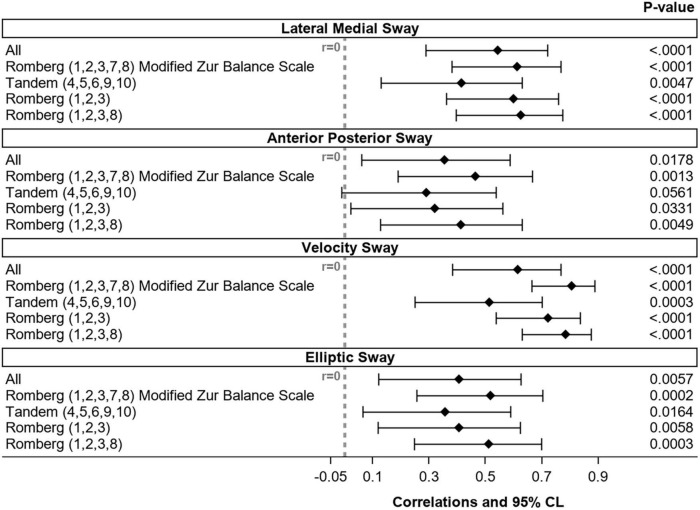
Correlation between the four kinematic parameters of the 10 ZBS tasks, as well as the Romberg and Tandem tasks and the mCTSIB (*N* = 44). CL, confidence limits.

Cronbach’s alpha (standardized values) to measure internal consistency of the 10 tasks of the ZBS score, are presented in Table 4 and Figure 5. It was < 0.75 for the M/L sway, A/P sway, and elliptical sway kinematic parameters for all 10 tasks, as well as for the Romberg and tandem tasks. Velocity sway on the other hand had a high alpha index of 0.84 for all 10 tasks, which increased to 0.85 when the tandem tasks were removed (α = 0.66). The highest alpha index was achieved with only three of the Romberg tasks.

**TABLE 4:** Cronbach’s standardized alpha for the 4 kinematic parameters of the 10 ZBS tasks, and the Romberg and Tandem tasks only (*N* = 44).

Task	M/L sway	A/P sway	Elliptical sway	Velocity sway
All 10 tasks	0.681	0.707	0.741	0.843
Romberg tasks (1, 2, 3, 7, 8)	0.573	0.671	0.667	0.840
Romberg tasks (1, 2, 3, 8)	0.541	0.682	0.691	0.853
Romberg tasks (1, 2, 3)	0.428	0.627	0.647	0.862
Tandem tasks	0.372	0.535	0.528	0.662

There was a positive correlation between the ZBS and the mCTSIB scores for the four kinematic parameters. The correlation was higher for the Romberg tasks of the velocity sway parameter, which is consistent with what was observed for the internal validity. This indicates that the tasks that contribute the most to the ZBS score are also those more strongly correlated with the mCTSIB.

### 3.5. Modified instrumental version of the ZBS (mZBS) and normative range

Interestingly, the correlation was highest between the five Romberg tasks, but Cronbach’s alpha was higher for only three of the Romberg tasks (Table 4 and Figure 5). Therefore, we decided that the modified ZBS (mZBS) would consist of the five Romberg tasks (1, 2, 3, 7, 8) (Table 5 and Appendix 1).

**TABLE 5:** Descriptive statistics of the 4 kinematic parameters for the mZBS (Romberg tasks) and normal ranges: overall and by age group (< 55, ≥ 55) (*N* = 44).

Parameter		M/L sway	A/P sway	Elliptical sway	Velocity sway
Romberg modified Zur Balance Scale	Mean (SD)	2.87 (0.57)	3.43 (0.80)	2.79 (0.60)	7.34 (2.90)
	Median [range]	2.88 [1.75; 3.99]	3.34 [2.25; 5.47]	2.76 [1.63; 4.14]	6.72 [3.14; 15.36]
	Normal range	[1.81; 3.81]	[2.30; 5.32]	[1.99; 4.01]	[3.21; 13.57]
Age group < 55	Mean (SD)	2.71 (0.53)	3.20 (0.66)	2.61 (0.52)	6.47 (2.49)
	Median [range]	2.67 [1.75; 3.81]	3.02 [2.25; 4.58]	2.48 [1.63; 4.01]	6.23 [3.14; 13.57]
	Normal range	[1.75; 3.81]	[2.30; 5.32]	[1.63; 4.01]	[3.14; 13.57]
Age group ≥ 55	Mean (SD)	3.28 (0.47)	4.02 (0.85)	3.29 (0.55)	9.68 (2.68)
	Median [range]	3.30 [2.33; 3.99]	3.97 [2.73; 5.47]	3.17 [2.36; 4.14]	9.33 [5.65; 15.36]
	Normal range	[2.33; 3.99]	[2.73; 5.47]	[2.36; 4.14]	[5.65; 15.36]

## 4. Discussion

The goals of the current study were to assess the inter- and intra-tester reliability of the ZBS, determine the normal ZBS scores for people ages 29–70, and validate the ZBS in comparison to the mCTSIB, using kinematic measurements.

The inter-tester and intra-tester reliability of the ZBS were high. Therefore, different testers can use the scale at different occasions, for primary evaluation and for reevaluating a patient’s balance function during rehabilitation. Both inter-tester and intra-tester MDCs of the total ZBS score were over 4.6 points, meaning that changes from 4.6 points can be considered clinically meaningful. Interestingly, the calculated MDC is very similar to that observed in clinical practice, as approximately 5 points.

This study was conducted with healthy individuals, who did not have balance deficits. We observed that the distribution of the normal scores decreased with age, which indicates that the ZBS is sensitive to differences in balance performance.

The greatest decrease in the ZBS scores occurred after age 60. These outcomes confirm information reported in the literature, where “older” is defined as above the age of 60 (Imms and Edholm, 1981; Woollacott et al., 1986). The effects of age on postural control mechanisms and on the vestibular system are well-documented (Inglin and Woollacott, 1988; Horak et al., 1989; Baloh et al., 2001).

The ZBS is used in the clinic to measure patients’ balance for a duration of 10 s. It contains challenging tasks that make a loss of balance noticeable. A force plate provides accurate kinetic data, which indicates that five Romberg tasks of the ZBS alone are sufficient for tracking changes in postural control. All participants were able to stand still in the Romberg position for the duration of the entire task, which also allowed better analysis of the sway data. When using kinetic measurements, the modified version of the ZBS—the mZBS should be used.

The results of this study indicate that the mZBS is as least as good as the mCTSIB for evaluating kinetic sway parameters of postural control in healthy adults, ages 29–70. The mZBS was found to be as valid and as reliable as the mCTSIB. The mCTSIB and the mZBS are both important tools for therapists assessing balance. However, the added benefit of the mZBS is that it focuses on the dynamic function of the vestibular system. Thus, vestibular impairments may be more easily identified by using the mZBS. This should be investigated further with vestibular patients. These findings correspond with previous results that found that the ZBS is as good as the Berg balance test for measuring balance in older adults, ages 71–97 (Zur et al., 2016).

There is a variety of clinical tests to measure postural stability. However, to the best of our knowledge, the ZBS is the only one that assesses postural stability with head movements while standing, and thereby challenges postural control with respect to all three-balance systems—visual, vestibular, and somatosensory. The main advantage of using the ZBS instead of the Berg Balance Scale (Berg, 1989), the timed up and go test (Whitney et al., 2004), or the functional reach test (Duncan et al., 1990) is that it assesses the vestibular system, which those tests do not evaluate at all. The advantage compared to the Mini BESTest (Horak et al., 2009), the Fullerton Advanced Balance (Rose et al., 2006) and the dynamic gait index (DGI) (Whitney et al., 2004) is that the ZBS does not need a walking track or large equipment, as it is performed when standing still on the floor and using a semi-circular piece of Styrofoam, 60 cm long×18 cm wide×9 cm high. The mCTSIB is also a suitable test, but it does not directly test the dynamic function of the vestibular system. Although the ZBS tests static and dynamic postural stability during quiet standing only, it can provide a good estimate of postural control, with an emphasis on the vestibular system.

## 5. Conclusion

The inter-and-intra tester reliability for the ZBS was high. The ZBS is quick to administer, scores are easily calculated, and it is suitable for a wide range of age groups.

Zur Balance Scale normal scores decrease with age, so the test can be used to evaluate even mild postural impairments at any age. It is an important tool for assessing postural stability and the effect of head movements on this stability. Additional studies are needed to determine the normal scores for children and adolescents.

## Data availability statement

The original contributions presented in this study are included in the article/supplementary material, further inquiries can be directed to the corresponding author.

## Ethics statement

The studies involving human participants were reviewed and approved by the Ethics Committee of the Institutional Review Board of the University of Haifa (approval no. 318, 319/16, date 12/09/2016). The patients/participants provided their written informed consent to participate in this study.

## Author contributions

OZ and HB-R: conceptualization, investigation, and data curation. OZ and EC: methodology. OZ, HB-R, and EC: validation. LD: formal statistical analysis. OZ: resources, visualization, project administration, and developed the Zur Balance Scale. OZ, HB-R, and LD: writing—original draft preparation. EC: writing—review and editing. All authors have read and agreed to the published version of the manuscript.

## Appendix 1

**Appendix Table A1:** ZBS score sheet.

Cond	Task	Head movements	Time (sec)
1	Romberg stance on the floor, eyes closed	X	
2	Romberg stance on the floor, during horizontal head movements, eyes open		
3	Romberg stance on the floor, during vertical head movements, eyes open		
4	Tandem stance on the floor, eyes open	X	
5	Tandem stance on the floor, eyes closed	X	
6	Tandem stance on the floor, during horizontal head movements, eyes open		
7	Romberg stance on the Styrofoam, eyes open	X	
8	Romberg stance on the Styrofoam, during vertical head movements, eyes open		
9	Tandem stance on the Styrofoam, eyes open	X	
10	Tandem stance on the Styrofoam, during horizontal head movements, eyes open		
	Score calculation (by number of head movements and seconds)	Max = 50 Head movements score	Max = 100 Total seconds score
Total	Final score = (Num*2+Sum in sec)/2	Num*2	Sum in sec
